# Repressive regulatory function of seryl-tRNA synthetase on *VEGFA* gene expression is impaired in renal cell carcinoma

**DOI:** 10.3325/cmj.2026.67.72

**Published:** 2026-04

**Authors:** Maja Barači, Petar Ozretić, Marijana Knezovic Florijan, Nika Foglar, Željko Kaštelan, Neda Slade, Jasmina Rokov-Plavec, Tvrtko Hudolin

**Affiliations:** 1 Department of Chemistry, Faculty of Science, University of Zagreb, Zagreb, Croatia; 2 Division of Molecular Medicine, Ruđer Bošković Institute, Zagreb, Croatia; 3 University Psychiatric Hospital Sveti Ivan, Zagreb, Croatia; 4 Genos Glycoscience Research Laboratory, Zagreb, Croatia; 5 Department of Urology, University Hospital Center Zagreb, Zagreb, Croatia

## Abstract

**Aim:**

To assess the relationship of the mRNA and protein expression levels of seryl-tRNA synthetase (SerRS) and the expression of gene encoding vascular endothelial growth factor A (VEGFA) in patients with renal cell carcinoma (RCC).

**Methods:**

Expression levels of *VEGFA* and *SerRS* mRNA were quantified by quantitative real-time polymerase chain reaction in 31 paired RCC tumor and adjacent healthy kidney tissues, while SerRS protein levels were assessed by western blot analysis in 19 paired samples. The association of SerRS and *VEGFA* with clinicopathological parameters was evaluated. In addition, bioinformatics analyses were performed using publicly available transcriptomic and proteomic data sets.

**Results:**

*VEGFA* expression was significantly higher in tumor tissues than in adjacent healthy tissues, while *SerRS* mRNA levels were similar in both. However, SerRS protein was significantly elevated in tumor tissues. Despite elevated levels of SerRS protein in RCC tumor tissues, SerRS repressive regulatory function on *VEGFA* was impaired. This dysregulation was associated with increased SerRS phosphorylation, upregulation of several transcriptional activators, and hypomethylation of the *VEGFA* promoter – factors likely contributing to *VEGFA* overexpression. Importantly, higher *SerRS* gene expression was significantly associated with improved overall survival in patients.

**Conclusion:**

The findings indicate that various factors diminish SerRS repressor function leading to the *VEGFA* upregulation in RCC. Survival analysis suggests that SerRS may serve as a valuable prognostic biomarker and represent a potential therapeutic target in RCC.

Renal cell carcinoma (RCC) is a heterogeneous disease and the most common type of kidney tumor in adults, associated with a high mortality rate (1,2). Over the past decades, the incidence of RCC has increased, largely due to the widespread use of imaging techniques leading to incidental tumor detection (3). The most prevalent histological subtype of RCC is clear cell RCC (ccRCC), accounting for 70%-90% of the cases (4). A frequent and early molecular event in RCC development is the loss-of-function alteration in the von Hippel-Lindau (*VHL*) tumor suppressor gene (5). Functional inactivation of the VHL protein leads to stabilization and accumulation of hypoxia-inducible transcription factors HIF-1α and HIF-2α, resulting in the overexpression of downstream target genes, including *VEGFA*, which encodes for vascular endothelial growth factor A (VEGFA) (5). Increased levels of VEGFA protein promote excessive angiogenesis, which supplies tumors with oxygen and nutrients, thereby supporting tumor growth and metastasis (6). Notably, elevated VEGFA mRNA and protein expression is commonly observed in RCC (7-9).

When diagnosed early, RCC can often be effectively treated with surgical or ablative approaches (10,11). However, approximately one-third of patients present with or develop metastatic disease (10,12). The five-year survival rate exceeds 90% for early-stage RCC but drops below 20% for late-stage metastatic cases (1). Targeted molecular therapies have improved clinical outcomes in some patients with metastatic RCC (13). Therapeutic options include immune checkpoint inhibitors, mammalian target of rapamycin (mTOR) inhibitors, VEGF pathway inhibitors, and HIF-2α inhibitors (12-14). Despite advances in multimodal cancer therapies, the treatment of advanced RCC remains challenging due to the development of drug resistance and therapy-related side effects (12,15). Therefore, it is essential to elucidate the molecular mechanisms underlying RCC development and progression, and to identify new diagnostic and prognostic biomarkers, as well as effective therapeutic targets.

Aminoacyl-tRNA synthetases (aaRSs) are ubiquitous and essential enzymes that catalyze the attachment of amino acids to their cognate tRNAs, supplying aminoacyl-tRNAs for protein biosynthesis (16,17). Beyond their canonical role in translation, aaRSs exhibit remarkable functional versatility and have been implicated in a variety of human diseases, including cancer (18-22). Aspartyl-tRNA synthetase and cysteinyl-tRNA synthetase have been analyzed in more detail in ccRCC (23,24).

Seryl-tRNA synthetases catalyze the attachment of amino acid serine to tRNA^Ser^ (25). In addition to their canonical role, they perform alternative functions in other cellular processes, such as regulation of gene expression and stress responses (26-34). The human cytosolic seryl-tRNA synthetase (SerRS/SARS1), which is also localized to the nucleus, regulates fundamental biological processes such as angiogenesis, telomere maintenance, lipid metabolism, and signaling pathways (26,35-42). These alternative functions of SerRS are largely mediated by its protein-protein interactions (26).

In the nucleus, SerRS acts as a negative regulator of *VEGFA* transcription. It exerts its repressive function either alone or in complex with proteins such as SIRT2 and YY1, counteracting transcriptional activators including c-Myc, MAX, NFκB1, TFAP2A, SP1, and EGR1. This balance is essential for proper vascular development and postdevelopmental angiogenesis (35-37). However, post-translational modifications can inhibit the anti-angiogenic function of SerRS. Under hypoxic conditions, ATM/ATR-mediated phosphorylation of SerRS prevents its binding to the *VEGFA* promoter, enabling increased recruitment of c-Myc and HIF-1α, which leads to enhanced VEGFA mRNA and protein levels and angiogenesis (43). Similarly, nutrient deprivation induces glycosylation of SerRS, reducing its nuclear localization and promoting VEGFA mRNA and protein upregulation and angiogenesis (37).

SerRS is considered a potential tumor suppressor due to its anti-angiogenic properties and its ability to suppress multiple tumor-promoting pathways, including angiogenesis, de novo lipid synthesis, Wnt signaling, and telomere elongation (37,39-41,43). Interestingly, in some instances, SerRS has exhibited tumor-promoter characteristics by activating the PI3K-AKT-mTOR signaling pathway (42). Although SerRS has been identified as a potential tumor target, its potential role in RCC has not yet been investigated.

Given that SerRS is a key repressor of *VEGFA* transcription in various human cell lines, this study sought to determine whether the SerRS-mediated regulation of *VEGFA* was preserved in RCC tumor tissues. By integrating experimental data with bioinformatics analysis, we aimed to identify the factors contributing to *VEGFA* dysregulation in RCC. Considering that SerRS overexpression inhibits multiple tumor-promoting pathways, the potential of SerRS as a biomarker and therapeutic target in RCC was evaluated using patient survival analysis.

## PATIENTS AND METHODS

### Patients and surgical specimens

Thirty-one randomly selected patients with primary, previously untreated RCC and complete clinico-pathohistological data were retrospectively assessed at the University Hospital Center Zagreb, Croatia (44). All tumors were histologically confirmed as RCC (all RCC cell types were eligible) by a clinical pathologist. Patients older than 18 years, with all stages of disease according to the TNM classification system, were included in the study. None of the patients had received radiotherapy or chemotherapy before surgery. After radical or partial nephrectomy, tumor specimens and corresponding adjacent non-tumor tissues were frozen in liquid nitrogen and then stored at −80 °C for RNA and, if available, for protein extraction. Samples were collected from patients who underwent radical or partial nephrectomy between November 2010 and October 2013. This study complied with the Helsinki Declaration and was approved by the Ethics Committee of the University Hospital Center Zagreb.

### RNA extraction and RT-qPCR

Total RNA was isolated from paired tumor and adjacent healthy control tissue samples from each patient using the TRIzol Reagent (Thermo Fisher Scientific, Waltham, MA, USA) according to the manufacturer’s instructions. Extracts were purified using the RNeasy Micro Kit (Qiagen, Germantown, MD, USA), including DNase I treatment, according to the manufacturer’s protocol. Total RNA was quantified and its purity assessed using the BioSpec-nano Micro-volume UV-Vis Spectrophotometer (Shimadzu, Kyoto, Japan). RNA was reverse transcribed using the High Capacity cDNA Reverse Transcription Kit (Thermo Fisher Scientific) according to the manufacturer’s instructions. Quantitative real-time polymerase chain reaction (PCR) was performed on a 7900 Real-Time PCR System (Applied Biosystems, Waltham, MA, USA) using Power SYBR Green PCR Master Mix (Applied Biosystems) according to the manufacturer’s instructions, with the reaction volume adjusted to 25 μL. Prediluted (1:5) cDNA was used for quantification of reference gene and target gene expression. Cycling conditions were as follows: denaturation at 95 °C for 10-minute, followed by 40 cycles at 95 °C for 15 seconds and at 60 °C for 60 seconds. The relative mRNA levels were normalized to TATA box-binding protein (TBP) using the 2^-ΔΔCt^ method (45). *TBP* is a suitable reference gene for gene expression studies in RCC (46). Primer pairs for *SerRS* and *VEGFA* genes were designed using NCBI Primer-BLAST (https://www.ncbi.nlm.nih.gov/tools/primer-blast/) (47). *TBP* primers sequences were taken from Latil et al (48). The following primers were used: for *TBP,* 5′- CAC GAA CCA CGG CAC TGA TT −3′ (forward) and 5′- TTT TCT TGC TGC CAG TCT GGA C −3′ (reverse), for *SerRS,* 5′- CCCAGAGAATGTGCTGAGTTTCG −3′ (forward) and 5′- CTCAAACCGCTCTGCTTCCAAC −3′ (reverse), and for *VEGFA,* 5′- TCT TCA AGC CAT CCT GTG TGC −3′ (forward) and 5′- CTT GGT GAG GTT TGA TCC GC −3′ (reverse).

### Protein extraction and western blot analysis

Approximately 100 mg of tissue specimens was homogenized for 25 seconds in 600 μL of ice-cold lysis buffer (50 mM Tris pH 8; 150 mM NaCl; 0.5 mM EDTA; 1% NP-40; 0.5% sodium deoxycholate; 0.1% SDS; 0.1% DTT; 100 μM sodium metavanadate) containing protease inhibitors. The homogenates were centrifuged for 30-minute at 12 000 g at 4 °C to remove cellular debris, and whole protein extracts were stored at −80 °C until western blot analysis. Protein concentrations were determined using the Bradford assay. Equal amounts of protein (20 μg) from healthy and tumor tissue samples were denatured before separation on 9% SDS-PAGE and transferred onto a nitrocellulose blotting membrane (Amersham Protran, Cytiva, Marlborough, MA, USA). Membranes were blocked with 5% non-fat dry milk dissolved in Tris-buffered saline (TBS) containing 0.2% Tween-20 (Merck, Rahway, NJ, USA) at room temperature for 1 hour, then thoroughly washed. Membranes were incubated overnight at 4 °C with primary antibodies against SerRS (1:1000; ab154825, Abcam, Cambridge, UK) and β-actin (1:1000; ab8227, Abcam). Membranes were washed three times with TBST for 10-minute each. Horseradish peroxidase-conjugated secondary anti-rabbit antibody was added at 1:2500 and 1:10 000 dilution for the detection of SerRS and β-actin, respectively, and membranes were incubated at room temperature for 1 hour. Afterwards, membranes were washed and prepared for detection. Signals corresponding to specific proteins on the membrane were developed by enhanced chemiluminescence using the ECL kit (Amersham ECL Select Western Blotting Detection Reagent, GE Healthcare, Buckinghamshire, UK) and detected on a C-DiGit Blot Scanner (LI-COR Biotechnology, Lincoln, NE, USA). Relative protein levels were determined using Image Studio Lite 5.0 (LI-COR Biotechnology) and normalized to β-actin. The specificity of SerRS band was confirmed using SerRS-His overexpressed from the pET20b_SerRS-His plasmid and purified by Ni-NTA chromatography. Total protein was not successfully extracted from several RCC tumor samples due to the inherent high fat/lipid content of the tumor cells, which can interfere with lysis methods. Additionally, paired samples for protein analysis were not available from all 31 patients. Therefore, SerRS protein analysis was performed on 19 paired healthy and tumor tissue samples.

### Statistical analysis of experimental data

The normality of data distribution was tested using the D’Agostino and Pearson omnibus test. Continuous variables were log-transformed before analysis to achieve a normal distribution. A paired *t*-test was used to assess differences in mRNA and protein expression levels between RCC and adjacent healthy tissues. Pearson correlation coefficient analysis was used to evaluate the correlation between mRNA and protein expression levels. A *t*-test was used to explore the association between clinicopathological variables and mRNA and protein expression. ANOVA with the Student-Newman-Keuls *post hoc* test was used to compare more than two groups. A two-tailed *P* < 0.05 was considered statistically significant. Statistical analysis was performed using MedCalc for Windows, version 17.6 (MedCalc Software, Ostend, Belgium).

### Bioinformatic analysis of public databases

Differential expression of selected genes, correlation analysis, and patient survival analysis were assessed using the Gene Expression Profiling Interactive Analysis 2 (GEPIA2) web server (http://gepia2.cancer-pku.cn/) (49). GEPIA2 is a user-friendly and interactive online resource developed to comprehensively analyze RNA sequencing expression data from tumor and normal samples in The Cancer Genome Atlas (TCGA) and the Genotype-Tissue Expression (GTEx) projects. The gene name used in the search for SerRS-related data was SARS. Gene expression analysis included 523 ccRCC patient samples from the TCGA-Kidney Renal Clear Cell Carcinoma (KIRC) data set (50), which were compared with 100 normal samples (72 TCGA-KIRC normal and 28 GTEx kidney samples). The cutoff value for |log_2_FC| was 1. Spearman’s correlation analyses were performed separately with tumor samples (TCGA-KIRC data set) and normal samples (TCGA-KIRC normal and GTEx normal kidney data sets). Patient survival analysis was performed based on the TCGA-KIRC data set. Median mRNA expression values were used as the cut-off value for prognostic analysis. Hazard ratios, corresponding 95% confidence intervals, and Kaplan-Meier curves, compared with the log-rank test, were computed using GEPIA2.

Protein, phosphoprotein, and promoter methylation levels were assessed using the University of Alabama at Birmingham Cancer (UALCAN) data analysis portal (https://ualcan.path.uab.edu/) (51,52). UALCAN is a comprehensive, user-friendly, and interactive web resource for analyzing cancer omics data. Proteomics data were obtained from the Clinical Proteomic Tumor Analysis Consortium (CPTAC). The gene and protein name used in the search for SerRS-related data was SARS. The CPTAC data set 'Clear cell renal cell carcinoma (extended)' included 219 tumor and 169 normal samples (53). Promoter methylation data were obtained from the TCGA-KIRC data set, which included 324 tumor and 160 normal samples. In both GEPIA2 and UALCAN analyses, *P* < 0.05 was regarded as statistically significant.

## RESULTS

### Demographic, clinical, and histopathological features of the patient cohort

Paired clinical specimens were collected from 31 patients who underwent radical or partial nephrectomy. Among them, 27 patients (87.1%) were diagnosed with ccRCC, the most common histological subtype of kidney cancer. Approximately half of the patients (45.2%) exhibited symptoms of RCC (hematuria, lumbar pain, abdominal mass) or symptoms of metastatic disease, while other patients (54.8%) were asymptomatic and were diagnosed incidentally using imaging techniques. Clinical and histopathological characteristics of the patients are summarized in Table 1.

**Table 1:** Characteristics of the patient cohort

Variable	n	%
Age (years)		
median (range)	64	31-82
<60	12	38.7
60-70	8	25.8
>70	11	35.5
Sex		
male	17	54.8
female	14	45.2
Histological subtype		
clear cell	27	87.1
non-clear cell	4	12.9
Tumor size (cm)		
≤7	14	45.2
>7	17	54.8
TNM stage		
I	15	48.4
II	3	9.7
III	10	32.3
IV	3	9.7
Fuhrman grade		
1	0	0.0
2	8	26.7
3	13	43.3
4	9	30.0
Presence of symptoms		
absent*	17	54.8
present†	14	45.2
Renal capsular invasion		
negative	21	67.7
positive	10	32.3
Venous invasion		
negative	24	80.0
positive	6	20.0
Local invasion		
negative	20	64.5
positive	11	35.5
Perirenal fat invasion		
negative	22	73.3
positive	8	26.7
Lymph node metastasis		
negative	25	86.2
positive	4	13.8
Distant metastasis		
negative	28	90.3
positive	3	9.7

*asymptomatic patients who were diagnosed incidentally using imaging techniques.

†patients with symptoms of renal-cell carcinoma (hematuria, lumbar pain, abdominal mass) or symptoms of metastatic disease.

### Expression levels of VEGFA and SerRS mRNA and SerRS protein in tumor and normal tissues

**Figure 1:**
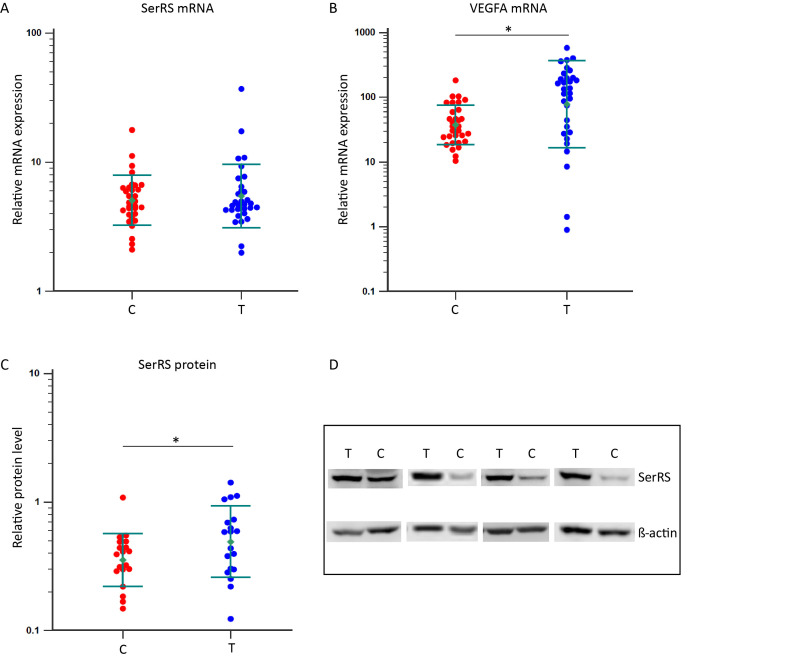
(**A**) Relative *SerRS* mRNA expression in renal cell carcinoma (RCC) and control healthy tissues evaluated by quantitative real-time polymerase chain reaction (RT-qPCR). (**B**) Relative *VEGFA* mRNA expression in RCC and control healthy tissues evaluated by RT-qPCR. Expression levels in (**A**) and (**B**) were normalized to the reference gene *TBP*. (**C**) Relative SerRS protein levels in RCC and control healthy tissues evaluated by western blot analysis. Results were normalized to β-actin. (**D**) Exemplary western blot detection of SerRS and β-actin in four paired samples. Plots show mean values ± standard deviations. C – control healthy tissue, T – RCC tissue. *P < 0.05.

*SerRS* and *VEGFA* expression levels were examined in 31 paired tumor and adjacent nontumor tissue samples using RT-qPCR (Figures 1A, 1B). Expression levels were normalized to *TBP*. *VEGFA* was significantly upregulated in tumor tissues compared with normal tissues (*P* = 0.018), while the groups did not differ in *SerRS* mRNA levels (*P* = 0.301). SerRS protein levels were analyzed by western blot in 19 pairs of matched tumor and healthy samples (Figure 1C). SerRS protein expression was significantly higher in tumor tissues compared with normal tissues (*P* = 0.033). There was a significant moderate positive correlation between *VEGFA* and *SerRS* mRNA levels in tumor tissues (R = 0.45, *P* = 0.011; Table 2).

**Table 2:** Pearson correlation coefficient (R) analysis between *VEGFA* mRNA, *SerRS* mRNA and SerRS protein expression in renal cell carcinoma (T) and healthy control (C) tissues. Statistically significant R is in bold

	SerRS (C)	*SerRS* (T)	*SerRS* (C)	*VEGFA* (T)	*VEGFA* (N)	
	R = 0.18	R = 0.00	R = −0.11	R = 0.20	R = 0.02	SerRS (T)
	*P* = 0.472	*P* = 0.994	*P* = 0.654	*P* = 0.419	*P* = 0.939	
		R = 0.15	R = 0.15	R = 0.40	R = 0.18	SerRS (C)
		*P* = 0.549	*P* = 0.540	*P* = 0.086	*P* = 0.461	
			R = 0.85	R = 0.45	R = 0.31	*SerRS* (T)
			*P* < 0.0001	*P* = 0.011	*P* = 0.088	
				R = 0.42	R = 0.29	*SerRS* (C)
				*P* = 0.020	*P* = 0.114	
					R = 0.20 *P* = 0.290	*VEGFA* (T)

### Association of VEGFA and SerRS with clinicopathological parameters

The associations of *VEGFA* mRNA, *SerRS* mRNA, and SerRS protein levels with various clinicopathological features were evaluated (Table 3). Several significant associations were observed: higher *VEGFA* mRNA expression was associated with local invasion (*P* = 0.021), lower *SerRS* mRNA expression was associated with the presence of symptoms (*P* = 0.009), while higher SerRS protein expression was observed in patients aged between 60 and 70 years (*P* = 0.029). However, no significant differences were found in expression levels relative to sex, tumor size, TNM stage, Fuhrman grade, renal capsular invasion, venous invasion, perirenal fat invasion, lymph node involvement, or the presence of distant metastases.

**Table 3:** Association of *VEGFA* mRNA, *SerRS* mRNA and SerRS protein expression with the clinicopathological parameters of patients with renal cell carcinoma

	*p-*values		
Parameter	*VEGFA*	*SerRS*	SerRS
Age (<60 vs 60-70 vs >70 y)	0.511	0.054	0.029
Sex (male vs female)	0.413	0.067	0.641
Tumor size (≤7 vs >7 cm)	0.398	0.592	0.375
TNM stage (I vs II vs III vs IV)	0.225	0.356	0.956
Fuhrman grade (2 vs 3 vs 4)	0.265	0.278	0.748
Presence of symptoms	0.844	0.009	0.676
Renal capsular invasion	0.099	0.381	0.769
Venous invasion	0.165	0.690	0.833
Local invasion	0.021	0.258	0.624
Perirenal fat invasion	0.070	0.298	0.969
Lymph node metastasis	0.919	0.925	0.399
Distant metastasis	0.831	0.086	N.A.

### Bioinformatics analysis of VEGFA and SerRS in ccRCC

**Figure 2:**
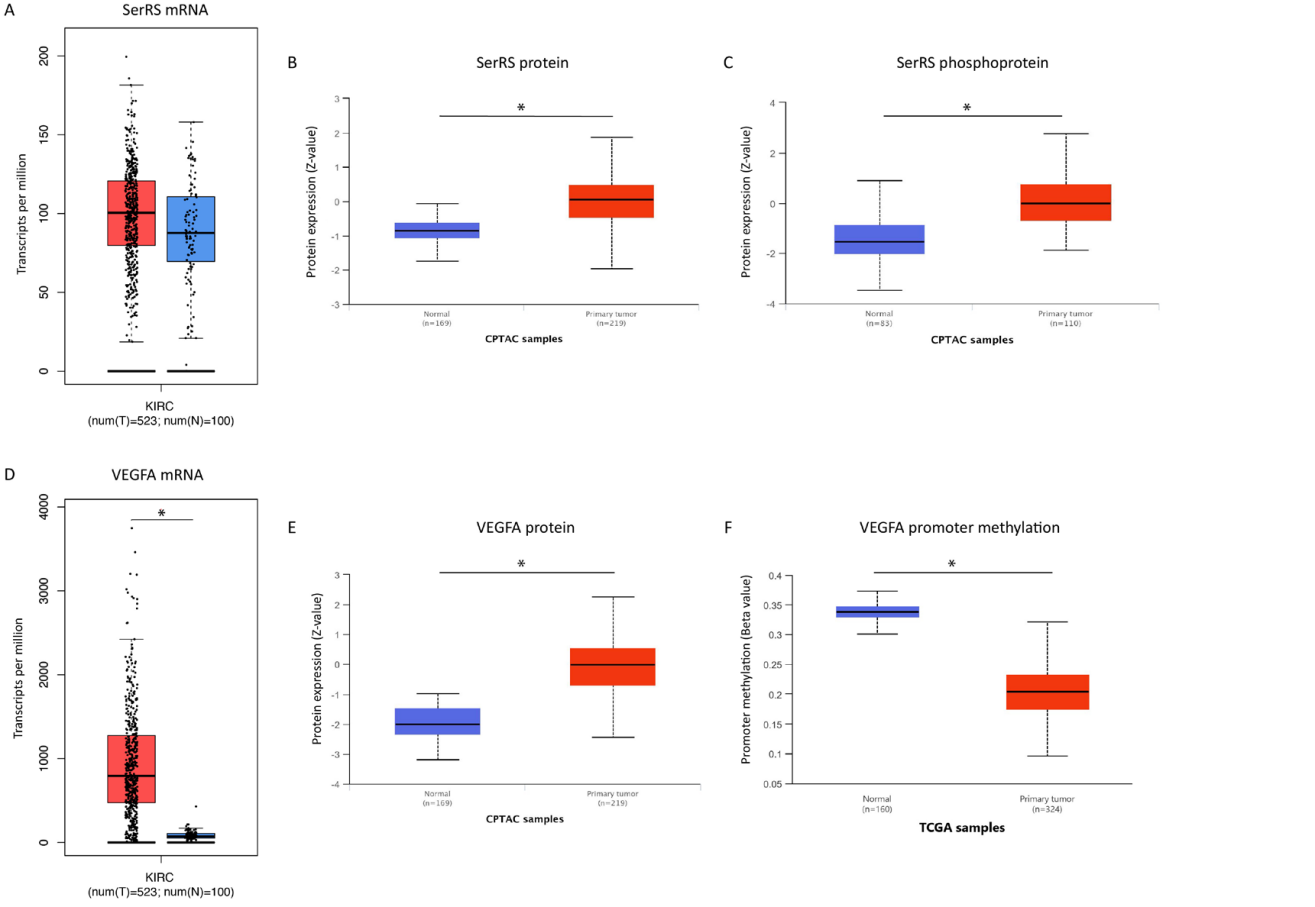
Bioinformatic analysis of publicly available data using Gene Expression Profiling Interactive Analysis 2 (GEPIA2) and University of Alabama at Birmingham Cancer (UALCAN) tools. Expression of *SerRS* (**A**) and *VEGFA* (**D**) genes in TCGA-Kidney Renal Clear Cell Carcinoma (TCGA-KIRC). TCGA-KIRC tumor data were matched to TCGA normal and Genotype-Tissue Expression (GTEx) data using GEPIA2. Expression of SerRS (**B**) and VEGFA (**E**) proteins in clear cell renal cell carcinoma. (**C**) Expression of SerRS phosphoprotein. Protein and phosphoprotein expression was analyzed in Clinical Proteomic Tumor Analysis Consortium (CPTAC) data set 'Clear cell renal cell carcinoma (extended)' using UALCAN. (**F**) *VEGFA* promoter methylation level in KIRC. The beta value indicates the level of DNA methylation ranging from 0 (unmethylated) to 1 (fully methylated). TCGA-KIRC tumor samples were matched to normal healthy samples using UALCAN. The box-whisker plots present interquartile ranges, including minimum, first quartile, median, third quartile, and maximum values. Normal healthy samples – blue, tumor samples – red. *P < 0.05.

**Figure 3:**
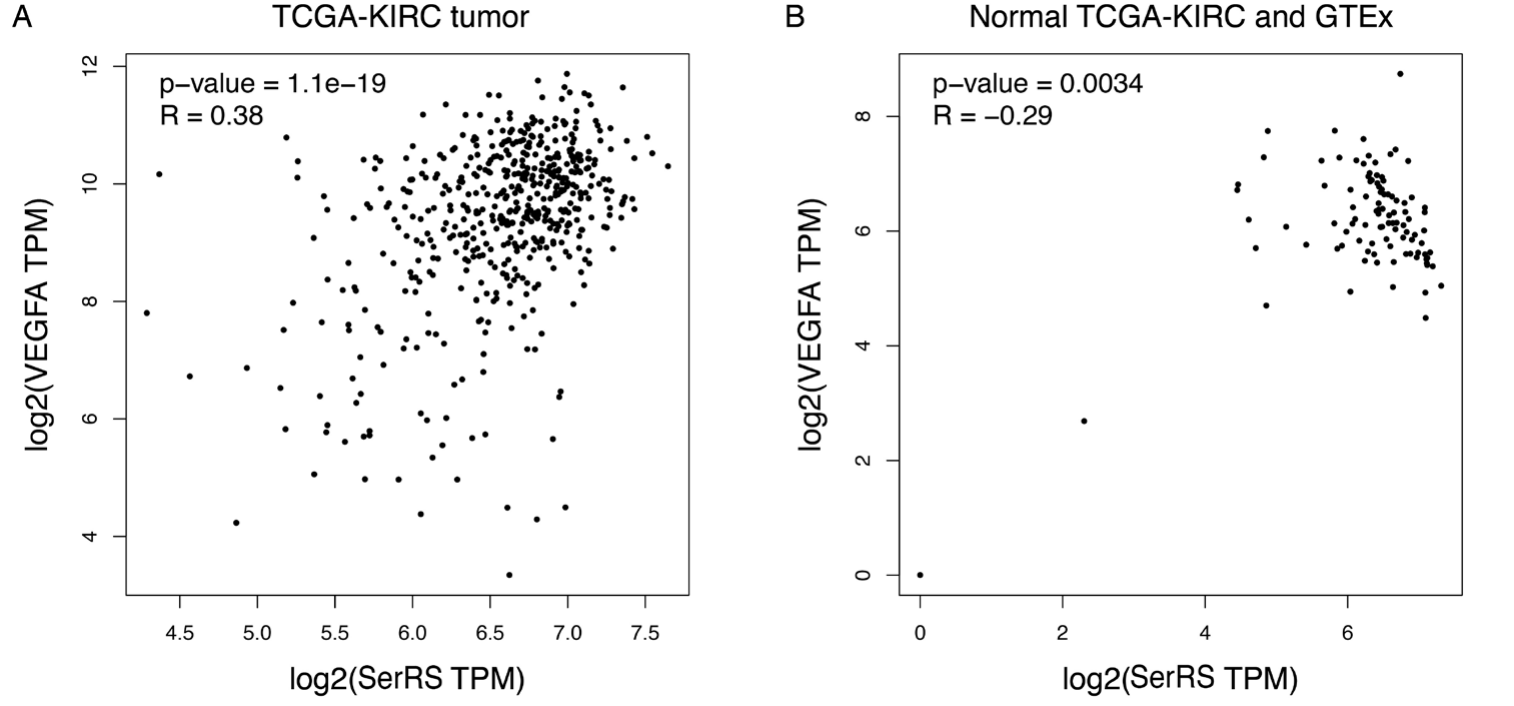
(**A**) Correlation of *SerRS* and *VEGFA* expression in TCGA-KIRC tumor samples. (**B**) Correlation of *SerRS* and *VEGFA* expression in healthy samples (TCGA-KIRC normal and GTEx). Graphs were generated using Gene Expression Profiling Interactive Analysis 2 tools. R - Spearman correlation coefficient. TPM – transcripts per million, TCGA – The Cancer Genome Atlas, KIRC – kidney renal clear cell carcinoma, GTEx – Genotype Tissue Expression database.

To complement our experimental findings, we performed bioinformatics analysis using publicly available cancer omics data. RNA sequencing expression data from TCGA-KIRC and GTEx kidney data sets were examined using GEPIA 2 tools. Consistent with our RT-qPCR results (Figure 1), *VEGFA* gene expression was significantly upregulated in tumors (Figure 2D), while *SerRS* mRNA showed no significant change (Figure 2A). Correlation analysis revealed a weak but significant positive correlation between *SerRS* and *VEGFA* mRNA expression in ccRCC tumors (R = 0.38, *P* = 1.1^−19^), consistent with our cohort data (Figure 3A). Interestingly, the correlation was weakly negative in normal (TCGA and GTEx) samples (R = −0.29, *P* = 0.003) (Figure 3B). Protein expression data from CPTAC data set ‘Clear cell renal cell carcinoma (extended)’ were analyzed via UALCAN data analysis portal. The SerRS protein was significantly increased in tumor compared with normal samples (Figure 2B), consistent with the results of our western blot analysis (Figure 1C). VEGFA protein was also significantly elevated (Figure 2E), in accordance with the upregulation of its gene expression (Figure 1B, Figure 2D), as shown previously (8).

### Overexpression of VEGFA despite increased SerRS protein – influence of posttranslational modification and transcriptional activators

**Figure 4:**
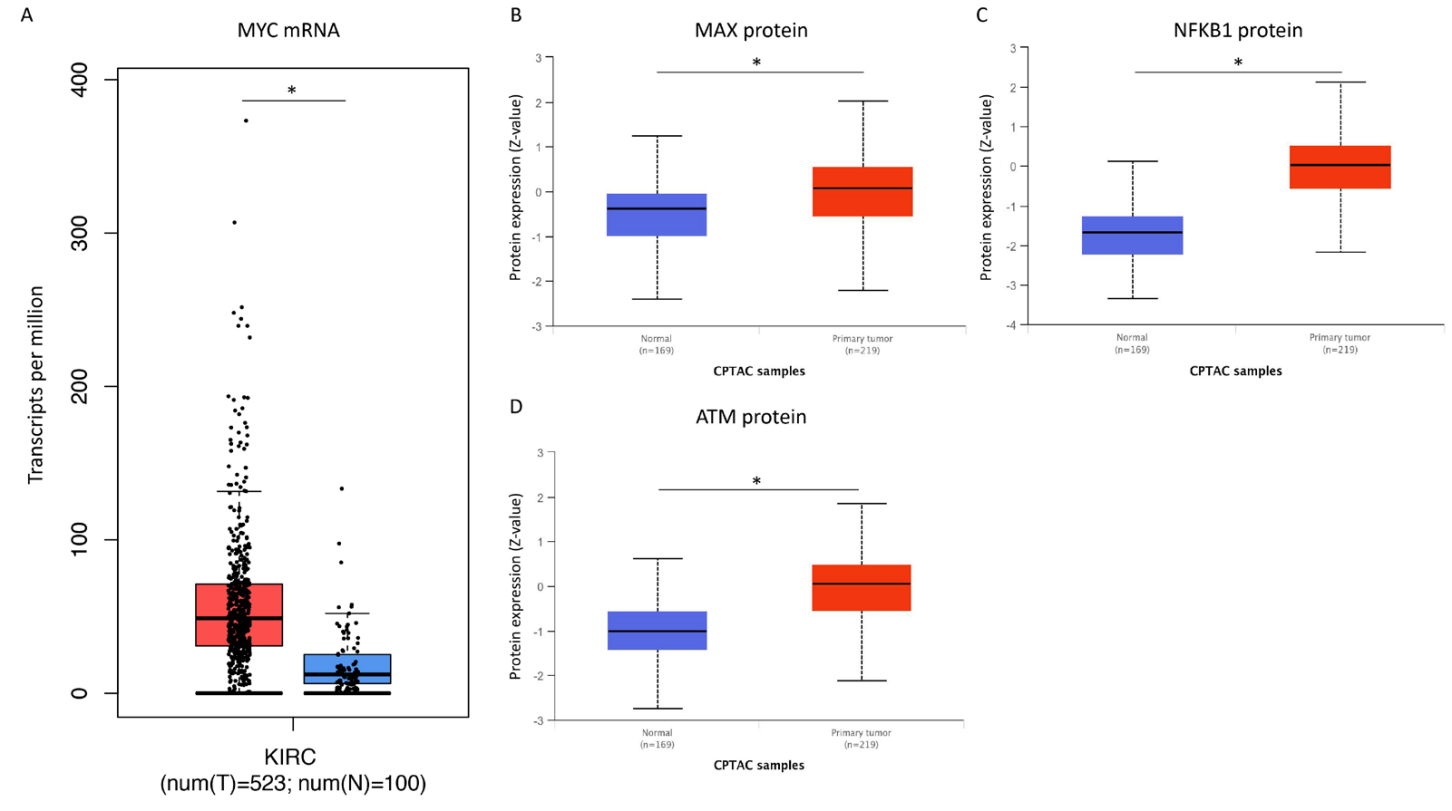
Differential expression of selected genes and proteins that may influence *VEGFA* expression. (**A**) Transcription factor MYC mRNA levels in KIRC. TCGA-KIRC tumor data were matched to TCGA normal and Genotype-Tissue Expression (GTEx) data using Gene Expression Profiling Interactive Analysis 2 (GEPIA2) tools. Protein levels of transcription factor MAX (**B**), transcription factor NFKB1 (**C**), and ATM protein-kinase (**D**) in clear cell renal cell carcinoma. Protein expression was analyzed using University of Alabama at Birmingham Cancer (UALCAN) database in the CPTAC data set 'Clear cell renal cell carcinoma (extended)'. The box-whisker plots present interquartile ranges, including minimum, first quartile, median, third quartile, and maximum values. Normal healthy samples – blue, tumor samples – red. KIRC – kidney renal clear cell carcinoma; CPTAC – Clinical Proteomic Tumor Analysis Consortium. * *P* < 0.05.

Previous research has demonstrated that SerRS protein acts as a transcriptional repressor for *VEGFA* gene expression and that overexpression of SerRS results in decreased VEGFA mRNA and protein levels in various cell lines (35-37,54-56). Intriguingly, our experimental data and bioinformatic analysis revealed that, in RCC, *VEGFA* mRNA levels were elevated despite an overexpression of SerRS protein. Furthermore, bioinformatics analysis also showed that VEGFA protein was elevated in RCC. The discrepancy between previous research and our findings may be explained by regulatory mechanisms specific for RCC, which diminish SerRS repressor activity or independently enhance *VEGFA* expression. SerRS repressor activity depends on its posttranslational modifications (37,43), while *VEGFA* gene expression is influenced by other factors besides SerRS protein (6). Therefore, we examined the expression of selected genes and proteins involved in these pathways using GEPIA2 and UALCAN (Figure 4). The transcription factor MYC was markedly upregulated at the mRNA level in tumor samples (Figure 4A). Protein levels of transcription factors MAX and NFKB1 were also significantly elevated (Figures 4B and C). ATM kinase, known to phosphorylate SerRS and inhibit its nuclear function, was significantly increased (Figure 4D). Indeed, by using UALCAN to analyze CPTAC phosphoproteome data, we found that the level of SerRS phosphoprotein was significantly higher in tumor samples than in normal samples (Figure 2C). Furthermore, we explored the methylation status of the *VEGFA* promoter, which can influence the epigenetic regulation of *VEGFA* expression. UALCAN analysis showed that the *VEGFA* promoter was significantly hypomethylated in TCGA-KIRC tumor samples compared with normal samples (Figure 2F). All these findings may account for the elevated VEGFA gene and protein expression despite the increase in SerRS protein in RCC.

### Association of SerRS and VEGFA gene expression with overall survival in ccRCC

**Figure 5:**
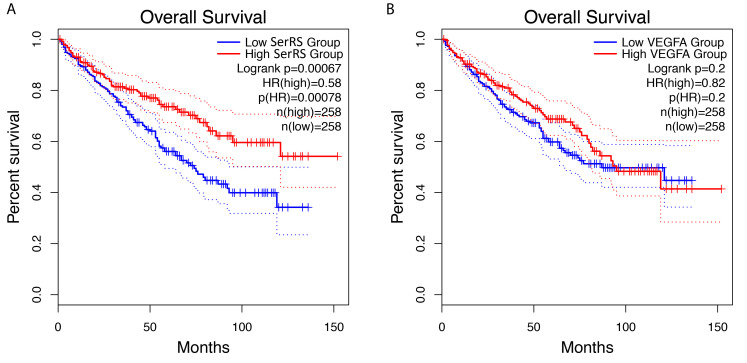
Relationship of *SerRS* (**A**) and *VEGFA* (**B**) expression with the overall survival of clear cell renal cell carcinoma patients. Kaplan-Meier survival curves based on mRNA expression from TCGA-KIRC data set were generated using Gene Expression Profiling Interactive Analysis 2 tools. The median expression values for *SerRS* and *VEGFA* were used to stratify samples into two groups: a high-expressing group (red) and a low-expressing group (blue). HR – hazard ratio, TCGA – The Cancer Genome Atlas, KIRC – kidney renal clear cell carcinoma.

To investigate the prognostic significance of *SerRS* and *VEGFA* gene expression in ccRCC, we analyzed overall survival data using GEPIA2. Patients were stratified into high and low expression groups based on median mRNA expression values. Kaplan-Meier survival analysis with log-rank testing revealed that higher *SerRS* expression was significantly associated with better overall survival (log-rank *P* = 0.0007) (Figure 5A). In contrast, *VEGFA* expression was not significantly associated with overall survival (Figure 5B).

## DISCUSSION

This study is the first to investigate the interplay between SerRS (SARS1) and VEGFA in RCC patients. *VEGFA* expression was significantly higher in tumor tissues compared with adjacent healthy tissues, while *SerRS* mRNA levels were similar in both. However, SerRS protein was significantly elevated in tumor tissues, which suggests posttranscriptional regulation. Surprisingly, despite the increase in SerRS protein, *VEGFA* mRNA levels were not reduced in RCC, indicating that SerRS repressive function in RCC was impaired. Bioinformatics analysis indicated that enhanced SerRS phosphorylation, increased levels of several transcriptional activators, and *VEGFA* promoter hypomethylation may contribute to *VEGFA* upregulation in RCC. Higher SerRS gene expression was associated with improved overall survival.

A hallmark of most RCC cases is the increased expression of the gene encoding VEGFA, a pivotal regulator of angiogenesis (7-9). Previous research on various cell lines has shown that several transcription factors activate *VEGFA*, while SerRS represses it to maintain balanced *VEGFA* expression, which is crucial for proper vasculogenesis and angiogenesis (6,35,36,57,58). However, posttranslational modifications like phosphorylation during hypoxia and glycosylation due to starvation can inhibit the repressive role of SerRS, leading to increased *VEGFA* expression, endothelial cell activation, and neovascularization (37,43).

Bioinformatics analyses using publicly available cancer omics data with GEPIA2 and UALCAN tools confirmed our experimental results: upregulation of *VEGFA*, similar *SerRS* mRNA levels, and increased SerRS protein in tumors compared with normal samples. Additionally, VEGFA protein was significantly elevated in RCC, consistent with upregulation of its gene expression. A positive correlation between *SerRS* and *VEGFA* expression in TCGA-KIRC tumor samples and a negative correlation in normal healthy samples further suggested inhibition of SerRS's repressive activity in RCC. Database analysis of the expression of various transcription factors, which were previously shown to compete with SerRS for the *VEGFA* promoter and activate *VEGFA* transcription (6,35,36), revealed that transcription factors MYC, MAX, and NFκB1 were significantly upregulated in ccRCC. Considering that phosphorylation of SerRS diminishes its binding to the *VEGFA* promoter (43), we analyzed SerRS phosphorylation status and protein levels of kinases that were known to phosphorylate SerRS (43). UALCAN analysis showed significantly upregulated ATM kinase, which aligns with increased levels of SerRS phosphoprotein in ccRCC observed in CPTAC phosphoproteome data. Furthermore, given that gene expression can be epigenetically regulated (59), methylation level of *VEGFA* promoter was determined. UALCAN analysis revealed that *VEGFA* promoter was significantly hypomethylated in tumor samples compared with normal samples, indicating transcriptional activation. Together, these findings suggest that posttranslational modification of SerRS, activation of pro-angiogenic transcription factors, and hypomethylation of *VEGFA* promoter may override the repressive function of SerRS, contributing to elevated *VEGFA* expression in RCC. Additionally, if SerRS nuclear localization is perturbed in RCC, this may also impact SerRS-mediated regulation of *VEGFA*.

The prognostic value of *SerRS* and *VEGFA* expression was assessed using the TCGA-KIRC data set. Higher expression of *SerRS* was associated with better overall survival outcomes for patients with ccRCC. A multi-omics study found that SerRS had tumor-suppressor-like properties in various human cancers and was one of the two most cancer-inhibiting aaRSs (22). Overexpression of *SerRS* has been shown to suppress tumor growth and development of metastasis in breast cancer models (41). Indicatively, high expression of *SerRS* is associated with better clinical outcomes in breast cancer patients (40). Our finding that *SerRS* overexpression in RCC is associated with improved patient survival indicates its potential as a prognostic marker and therapeutic target. Despite significant advancements in RCC research, a clinically actionable diagnostic or prognostic biomarker has yet to be identified (60,61). Treating RCC, particularly in advanced stages, remains challenging, as targeted molecular therapies often yield limited results (12). Therefore, discovering sensitive and specific biomarkers and exploring novel therapeutic targets in RCC is crucial. Several small molecules have been identified as inducers of *SerRS* expression, which could be explored for RCC treatment (54-56,62).

A limitation of our study is its retrospective design. As patient cohort size was modest, publicly available cancer omics databases were used to validate our experimental observations. Considering that higher levels of pro-angiogenic transcription factors, SerRS phosphoprotein, and *VEGFA* promoter methylation, observed in databases, were not additionally experimentally verified, the exact mechanism of inhibition of SerRS repressive role on *VEGFA* expression remains to be elucidated. Future studies should focus on validating the clinical value of *SerRS* as a biomarker and exploring the effectiveness of targeted therapeutic strategies for SerRS in the treatment of RCC.
